# Vena Cava Filters: Toward Optimal Strategies for Filter Retrieval and Patients' Follow-Up

**DOI:** 10.3389/fcvm.2022.746748

**Published:** 2022-03-03

**Authors:** Kiara Rezaei-Kalantari, David C. Rotzinger, Salah D. Qanadli

**Affiliations:** ^1^Rajaie Cardiovascular Medical and Research Center, Iran University of Medical Sciences, Tehran, Iran; ^2^Cardiothoracic and Vascular Division, Department of Diagnostic and Interventional Radiology, Lausanne University Hospital and University of Lausanne, Lausanne, Switzerland

**Keywords:** venous thromboembolism, IVC filter, retrieval strategies, complications, IVCF workflow

## Abstract

Mortality rates associated with venous thromboembolism (VTE) are high. Inferior vena cava filters (IVCFs) have been frequently placed for these patients as part of their treatment, albeit the paucity of data showing their ultimate efficacy and potential risk of complications. Issues regarding long-term filter dwell time are accounted for in society guidelines. This topic has led to an FDA mandate for filter retrieved as soon as protection from pulmonary embolism is no longer needed. However, even though most are retrievable, some were inadvertently left as permanent, which carries an incremental lifetime risk to the patient. In the past decade, attempts have aimed to determine the optimal time interval during which filter needs to be removed. In addition, distinct strategies have been implemented to boost retrieval rates. This review discusses current conflicts in indications, the not uncommon complications, the rationale and need for timely retrieval, and different quality improvement strategies to fulfill this aim.

## Introduction

Pulmonary embolism (PE) is the third most common cause of inpatient deaths (1), and anticoagulation (AC) is the mainstay treatment of it. IVC filters (IVCFs) were developed and are indicated to prevent fatal PE in therapeutic instances in which AC is not possible or efficient enough. IVCFs have gradually become a standard part of venous thromboembolism (VTE) management. A wide range of indications for IVCF placement has been considered during past decades, including therapeutic instances of AC contraindication, bleeding complications, inability to achieve or maintain optimal medical therapy or, rarely, treatment failure (2–4) as well as, certain prophylactic circumstances like multiple traumas or bariatric surgery.

There have been disagreements in societal guidelines recommendations for IVCF placement. These conflicts, while notifying the necessity for a consensus for optimal use, are sustained by two major factors: first, the scant convincing randomized data supporting the effectiveness of IVCF to prevent embolic events, improve outcomes and mortality; second, the significant increase of complications related to vena cava filters reported in the early 2000s. This review discusses the appropriateness, ideal timing, and challenges of IVCF retrieval.

## Rationale for Filter Retrieval

### Controversies in Vena Cava Filter Use

Some studies doubted the clinical benefit of IVCF in the eventual improvement of patient outcomes, which partly accounts for controversial variability of indications for IVCF placement between the centers (3, 5–11). A systematic review and meta-analysis of 11 studies on the effectiveness and safety of IVCFs comparing 2,055 patients who received a filter vs. 2,149 controls demonstrated that IVCF placement was associated with a 50% decline in the PE incidence and an about 70% rise in the deep vein thrombosis (DVT) risk over time. However, neither all-cause mortality nor PE-related mortality differed significantly between the two groups with or without filter placement (12). A recent retrospective cohort study also revealed no clinically meaningful reduced in-hospital all-cause mortality in stable patients under 80 years, constituting the primary candidates to IVC filtering (13). In their meta-analysis, Shariff et al. delineated that prophylactic use of IVCFs diminished the risk of symptomatic but non-fatal PE in major trauma patients (14). The PREPIC-2 trial randomized patients with PE and venous thrombosis to receive anticoagulant treatment, with or without a retrievable vena cava filter. In this trial, the rate of recurrent VTE did not differ between groups (7).

Current societal guidelines for filter placement do not concur on occasions. While the Society of Interventional Radiology (SIR) and American College of Radiology (ACR) guidelines state that a filter can be placed as prophylaxis for patients at high-risk of developing DVT or PE, the American College of Chest Physicians (ACCP) guidelines and the European Society of Cardiology (ESC) recommendations suggest IVCFs when the patient has an acute proximal lower extremity DVT with failure or intolerance to anticoagulants (15–18).

A joint study led by the Society for Vascular Surgery, the Society of Interventional Radiology, and the Food and Drug Administration (FDA) is ongoing. This multicenter, prospective, open-label, non-randomized investigation of commercially available IVCFs (retrievable and permanent) study looks into the Safety and Effectiveness of Inferior Vena Cava Filters (PRESERVE). IVCFs from seven manufacturers are under investigation to better understand the current use, safety, and effectiveness of IVCFs, and any adverse events associated with their use (19).

### Complications of Vena Cava Filters

Placement of an IVCF comes along with potential risks of complications. Such complications are not uncommon (Table 1) and can sometimes be grave. Several reports of filter complications include fracture and/or embolization and DVT that occasionally extends up to the IVCF (3, 20, 21, 25, 26).

**Table 1:** Complication rate reported with permanent and potentially retrievable devices in the literature (20–24).

Vascular access-related	4–11%
Filter fracture	1–2%
Filter migration	0–18%
Malposition	1–9%
IVC perforation	0–41%
VTE/PE	Up to 43%
IVC thrombus	2–30%
Embolization of filter fragments	<1%
Demise	0.12%

*VTE, venous thromboembolism; PE, pulmonary embolism*.

A systematic review revealed penetration of the venous wall in 19% of 9,002 procedures; of these, 19% showed near organ involvement, and more than 8% were symptomatic. Although fatal complications were uncommon, 5% of patients needed surgical removal of the filter or interventions such as endovascular stent placement or embolization, retrieval of the permanent filter, urinary diversion by percutaneous nephrostomy or even ureteral stent placement (27).

In a study assessing more than three hundred lawsuits pertaining to IVCFs, failure to prevent PE was the most common reported complication (28). Clinically significant VTE could happen despite IVCF presence due to ineffective filtration either because of migrated or tilted filter or due to thrombus propagation via a collateral vessel.

The MAUD database (21) clearly indicates that all filters are not equal in terms of IVCF-related long-term complications. Deso et al. (29) evaluated the reported IVC filter-related complications among filters of various geometries. They found a generalized tendency of higher fracture rates and perforation for conical filters and higher IVC occlusion with those having a cylindrical or umbrella-like morphology. Furthermore, some filter types like Optease and TrapEase filters were more often left in place compared to others, partly due to higher filter thrombosis and VTE recurrence under anticoagulant therapy (30, 31).

According to a retrospective study, complication rates have been higher among treated patients for prophylactic purposes than those receiving the filter for therapeutic purposes (32). This intensifies the call for more judicious placement of the filter for prophylactic purposes.

It should be kept in mind that the data disclosing IVCF-related complications might be underestimating the true prevalence since no routine follow-up imaging is usually performed, and a great portion of filter-related complications are “silent” (27).

The FDA approved retrievable IVCFs (rIVCFs) in the early 2000s to profit from the short-term benefits of filter placement without the associated potential long-term complications. Unless the rIVCF is removed, the same complications as permanent filters could happen. Even some retrievable filters are made of less durable material than their permanent counterparts, resulting in a potential risk of filter complication and fracture (21, 31). Analysis of the FDA Manufacturer and User Facility Device Experience (MAUDE) databased revealed that the majority of IVC filter complications were accompanied by retrievable IVC filters (86.8%) compared with permanent IVCF (13.2%) (21). Although, this could be justifiable to the more widespread use of the rIVCF's.

The mortality rate was higher for patients in whom filters were not retrieved (30). Nevertheless, despite the growing number of IVCF placements (31, 33), though most are retrievable, the retrieval rate reported in studies was only about 20–40% (20, 30, 34–37).

These limitations are reflected in FDA mandates (both in 2010 and 2014) which recommend that “implanting physicians and clinicians are responsible for the ongoing care of patients with retrievable IVCF and should consider removing the filter as soon as protection from PE is no longer needed.” (2, 38).

## Criteria for Filter Retrieval

### Clinical Eligibility

Primitive criteria for IVCF retrieval include the possibility of anticoagulant prescription if needed, an admissible retrieval-related embolism risk, and life expectancy more than 6 months, all with patients' desire and acceptance (20, 39).

On the other hand, certain situations preclude filter removal. These include patients' general condition like older age (>90 years), underlying disease (e.g., chronic renal failure), limited cardiopulmonary function and comorbidities (e.g., advanced malignancy, neurologic disorders), evidence of preoperative PE, anticoagulation failure after re-introduction, long-term contraindication of anticoagulation, and long-term immobilization and patient refusal (30, 40). If the patient presented with at least one clinical criterion on the day of placement, the filter would be declared permanent, and the follow-up visit is canceled. Otherwise, a re-assessment of clinical status will be done in a follow-up visit.

### Technical Eligibility

Imaging performed prior to IVCF retrieval either with CT venography (CTV) or peri-operative rotational venography (21, 41, 42) helps assess technical filter retrievability and plan the optimal approach and technique for filter removal. Although invasive assessment carries inherent potential risks, it but was proven more accurate in determining if the hook is embedded in the IVC wall, especially when using rotational venography (43, 44). An embedded hook is an important finding when planning the approach to IVC filter removal. In addition, CTV with the capability of depicting the perivascular components is helpful in demonstrating adjacent organ involvement in case of filter penetration, fracture and assessing the total extent of the clot burden.

**Figure 1:**
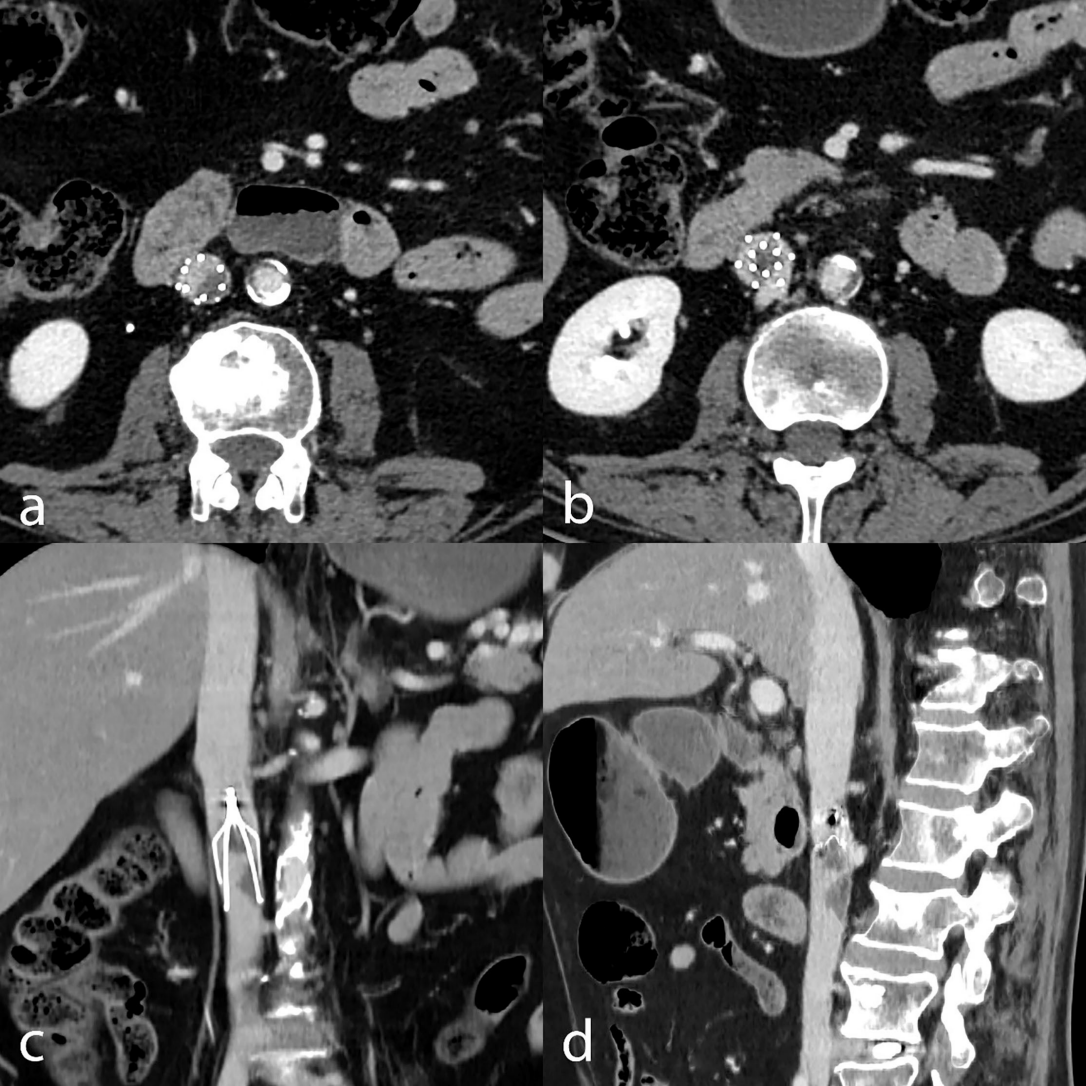
Technical irretrievability due to thrombus inside the filter. CT venography with axial **(a,b)**, coronally reformatted **(c)**, and sagittally reformatted minimum-intensity projection **(d)** images show thrombotic material stuck in the filter, precluding retrieval.

Technical irretrievability is considered in the presence of a large thrombus in the filter (>30% of filter volume, Figure 1) or deep transmural penetration. Although not considered a criterion of irretrievability, filter tilt is associated with increased technical unsuccessfulness (45).

## Optimal Time for Filter Retrieval

Depending on their manufacturer design and characteristics, IVCFs can be retrieved in a period ranging from weeks to several months.

It has been demonstrated that filter retrieval complication risk has been higher 6 months after placement in trauma patients (46). Further studies confirmed that post-procedure and retrieval complication rates correlated well to longer periods of dwell time, with rare occurrence within 30 days (31).

Therefore, IVCFs should ideally be retrieved as soon as possible. Though, in a practical view, a balance between the overall risks and benefits ought to be considered; the scheduled removal must be soon enough to stay within an acceptable time frame in terms of complications. On the other hand, removal should happen late enough to avoid recalling every patient who may still need the filter. A 5-month post-placement visit is an approach taken in the Lausanne University Hospital, Switzerland (Center Hospitalier Universitaire Vaudois or CHUV) to fulfill this aim (45).

## Current limitations of filter retrieval

**Figure 2:**
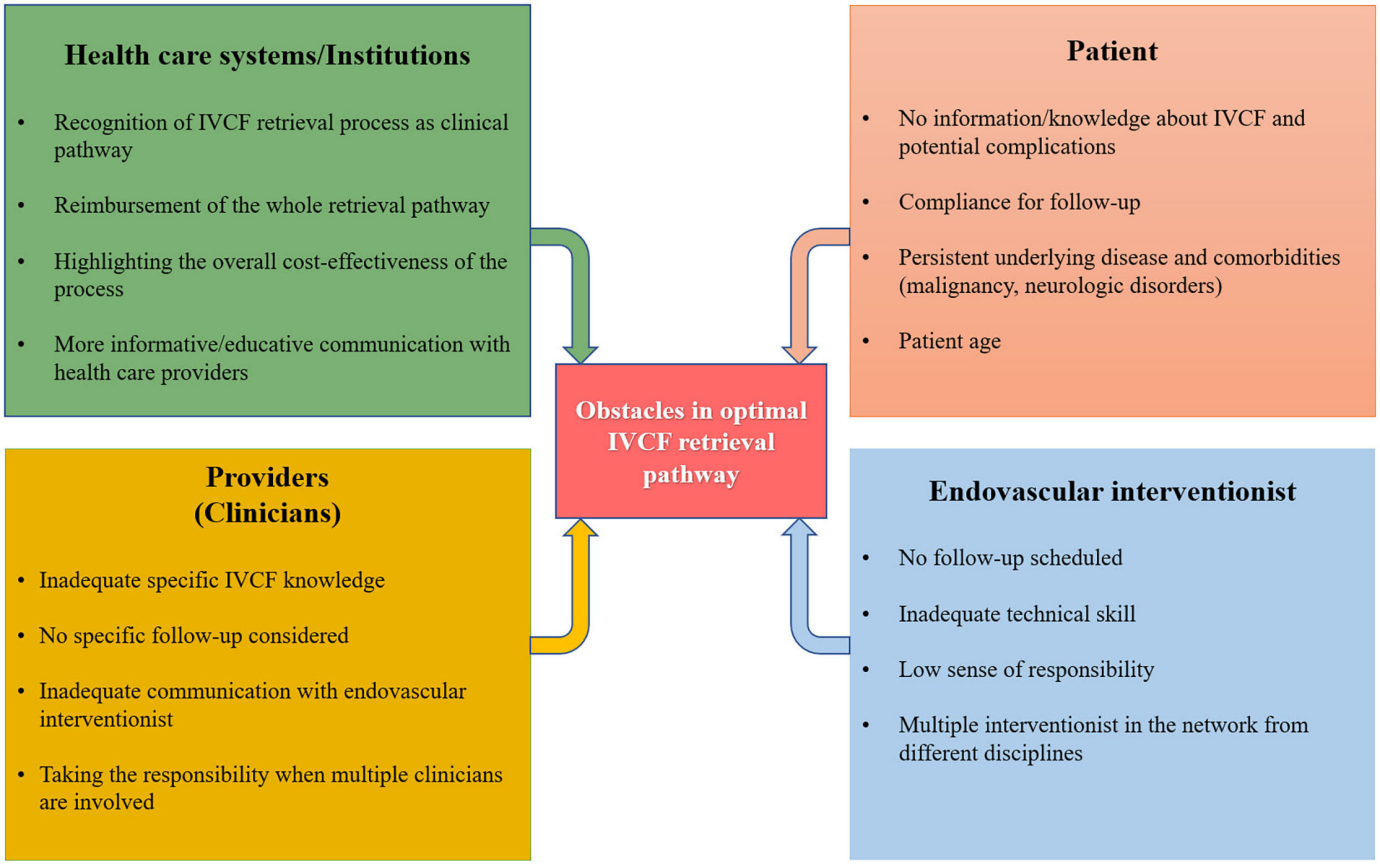
Brief review of the obstacles in IVC filter retrieval pathway.

Despite known complications, IVCFs are not retrieved frequently enough; rates ranging from 1 to 64% have been reported in the literature (30, 47–50). The main obstacle for rIVCF removal is the lack of follow-up of the patients, which is more problematic when the physician who placed the filter is not directly responsible for the follow-up (36). The problem becomes aggravated in the absence of a specifically designed IVCF follow-up program coinciding with the lack of efficient communication between the clinicians and interventional radiologists (IR), patient information or compliance, and sometimes IR responsibility (Figure 2).

**Figure 3:**
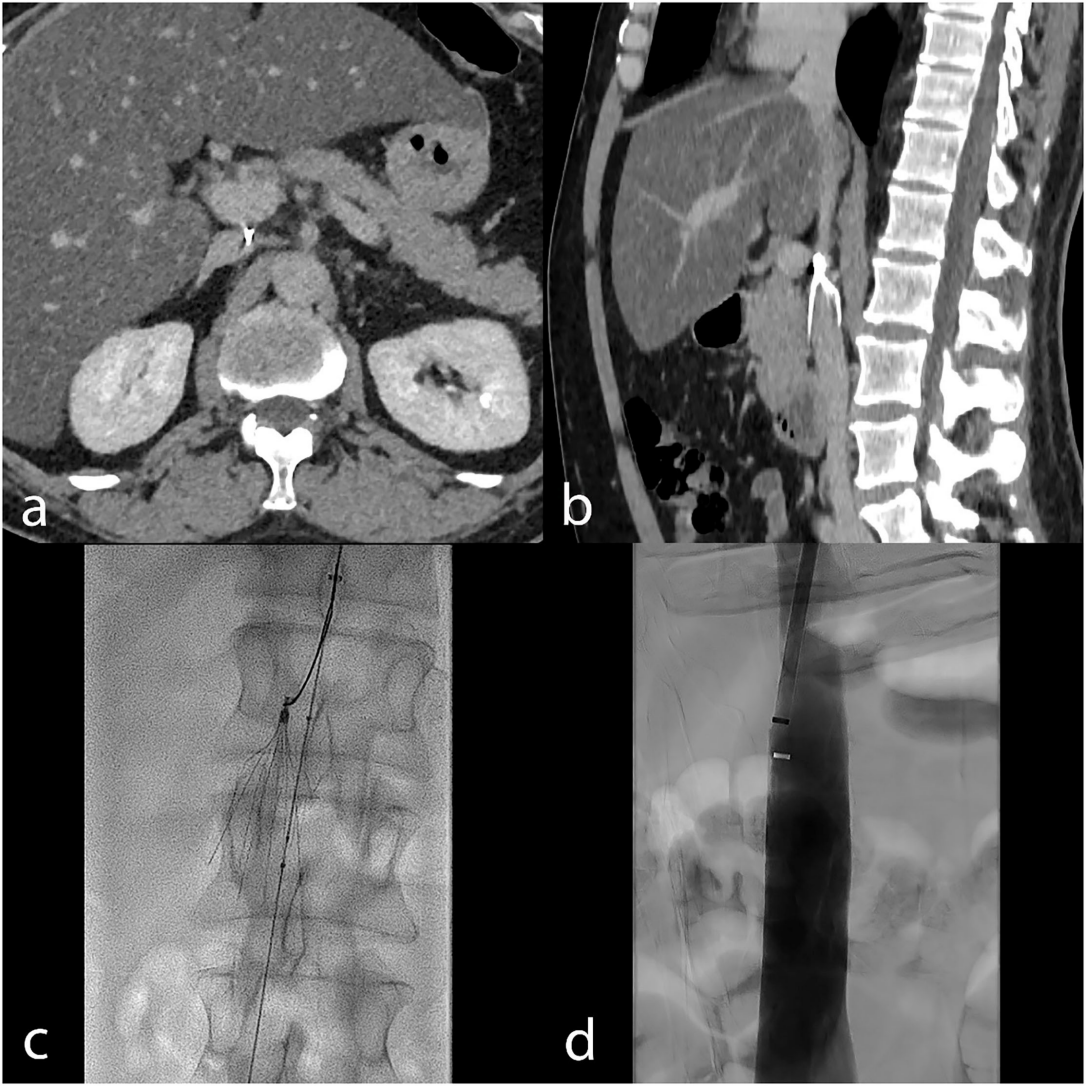
Embedded hook in the IVC wall is a common technical failure reason. CT venography in a patient with an embedded hook is shown on axial **(a)** and sagittal **(b)** images. In this case, advanced retrieval with the balloon displacement technique (periprocedural fluoroscopy image, **(c)** allowed removal. Final transcatheter subtracted cavography **(d)** shows the absence of complication.

The other issue is the technical success rate of the retrieval. Probing different technique reviews and studies which concerned IVC filter retrieval techniques, including the standard wire loop and snare, balloon displacement technique, dual access approach, forceps dissection, fibrin cap disruption, and thermal laser removal, reveals that about 40% to at best 84% of retrievable-type filters cannot be removed with standard retrieval techniques. The reason is that IVCFs either have become tightly embedded (Figure 3), are malpositioned or tilted (>15°) (40, 51–54). Other mentioned reasons for failure or difficult removal were significant strut penetration and prolonged dwell time (55) (Table 2). Filters with an embedded hook or that tilted >15° have been associated with 129 and up to 33 times higher risk of difficult retrieval, respectively (41, 56). In a study by Desai et al. (57), when filters were carried for more than 210 days, advanced retrieval techniques were needed in over 40% of the cases.

**Table 2:** Main reasons for standard retrieval technique failure and their overall incidence (40, 45, 53).

Filter embedded in IVC wall	4.7–6.2%
Filter angulation (>15°)	1.9–16%
Significant leg penetration (more than 15 mm)	0.3–2.7%
Prolonged dwell time (due to lack of follow-up)	10–29%

## Complications of Retrieval Procedures

**Figure 4:**
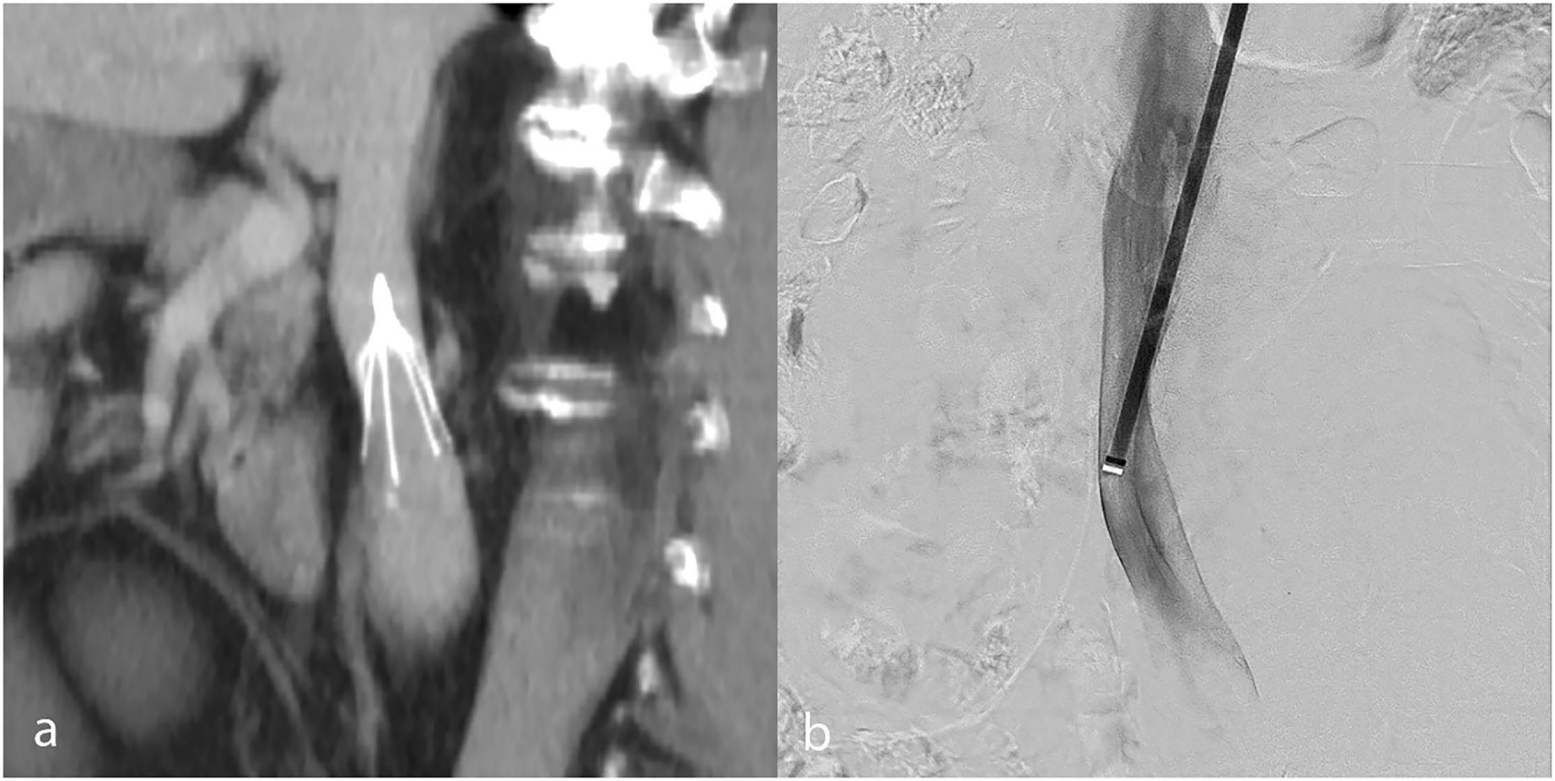
IVC stenosis due to IVCF use, shown in a patient on coronally reformatted pre-retrieval CT venography **(a)**. Post-retrieval subtracted catheter-directed cavography **(b)** confirms a persistent focal IVC stenosis at the former filter site.

Filter retrieval itself is not without a complication risk. Reported rates in studies vary from 0 to 20% (58, 59) and are influenced by different factors, including the technique used for removal and the filter position (37). Complication risk is significantly higher when advanced techniques other than simple snare removal are required (59, 60). Table 3 summarizes the major complication of the IVCF retrieval and their incidence stratified by the technique used (58, 60–68). Rare cases of cardiac tamponade secondary to filter retrieval has also been reported (69). Another possible complication is IVC stenosis following filter use (Figure 4).

**Table 3:** Major complication of IVCF retrieval and their reported rate stratified by the technique used.

**Retrieval technique**	**Major complications**	**Overall rate**
Standard wire loop and snare	Access complications (e.g., pneumothorax, jugular vein thrombosis)	0–5%
Forceps dissection	Leg fracture and embolization, IVC perforation, Renal artery to IVC fistula	0.8–11.8%
Laser thermal removal	IVC Thrombosis, IVC perforation	0–3%
Sling technique	IVC dissection, IVC perforation, leg fracture	0–20%

## Strategies to Optimize Retrieval Rates

Given the risk of complications associated with a longer IVCF dwell time, peculiar strategies have been implemented in centers to approach the issue (Table 4). A structured program for patient follow-up after IVCF placement is the primary step to take action when dealing with the issue.

**Table 4:** IVC filter retrieval strategies, advantages, and disadvantages.

	**Advantages**	**Disadvantages**
Clinical IR retrieval visit	Simple, practical, with cost-benefit. No need for dedicated staff or an extensive database. Could have widespread adoption by centers with different levels of health care	Individual-based
VTE team	Multidisciplinary decision-making, increases the interdisciplinary connection, more appropriate management of anticoagulation lowers the legal burden of patient management.	Time-intensive, needs commitment to maintaining efforts for continuous monitoring and follow-up
Dedicated IVCF clinic	Clinical database, establishes a standard method to coordinate the removal	Resource-intensive, Needs dedicated staff.
IVCF registry	Comprehensive database, theoretically has the highest success rate and lowest loss to-follow up	Sophisticated, time and resource-intensive, applicable in tertiary academic centers, needs dedicated staff

Some approached the question by organizing a VTE team, especially in tertiary care institutions, with clear documentation of an active decision-making process. This process involves the primary responsibility of the anticoagulation, IVCF indication, type, and time period as well as retrieval probability and indication (70–72). Others applied a multidisciplinary task-force (73) to implement a new IVCF retrieval protocol or a dedicated IVCF retrieval clinic (74) to build a comprehensive database prospectively.

These focused and determined dedicated IVCF strategies to track IVCF patients, although more sophisticated and resource-intensive, have effectively improved retrieval rates (74–78). Kalina et al. (76) demonstrated that the retrieval rate improved from 15.5 to 31.5% with the use of a “filter registry.” Another report by Sutphin et al., based on the Define, Measure, Analyze, Improve, Control (DMAIC) methodology of the Six Sigma process improvement paradigm, showed an increase in optional IVC filter retrieval rate from 8 to 40%. They identified the barriers to filter removal and grouped them into four categories: providers, patients, clinical, and systems. They stated that provider knowledge and communication, patient knowledge and follow-up after the procedure, lack of a formal patient database, and shortage of permanent filters in stock for patients who need it were the key barriers in their respective categories (78).

**Figure 5:**
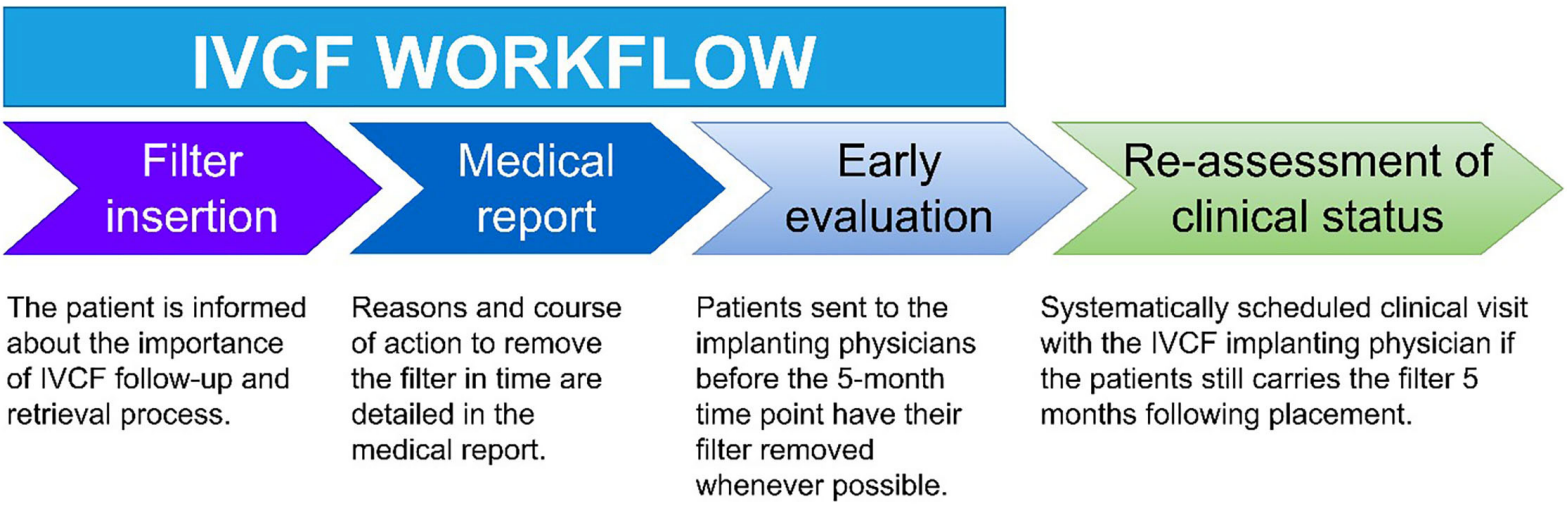
Recommended inferior vena cava filter (IVCF) management, such as implemented by the authors. A clinical follow-up visit at the 5-month time point is critical to achieving appropriate retrieval rates.

After placing a temporary IVCF, the procedure's report must explicitly address a follow-up plan and time limit to clarify the responsibility and ensure that timely retrieval will be attempted. This will ease a systematic follow-up visit at the scheduled time–at 5 months in the authors center—and give the dedicated IR staff liability to follow the patient (79). A copy of the medical report is sent to the patient's primary care physician, who is not necessarily aware of potential long-term IVCF-related risks. The report should contain instructions encouraging a follow-up IR visit whenever the filter is no longer needed. Furthermore, patients should be personally informed about the temporary nature of the device at the time of placement. Figure 5 summarizes the approach the authors recommend; special attention to see patients in a follow-up visit at the 5-month time point is crucial to ensure that IVCFs are retrieved if possible.

Clinical visits for follow-up could be conducted by a member of the interventional radiology (IR) department assigned to this task (IVCF IR referent), leading to a more similar and homogenous management process. This established quality improvement program led to about 48% additional retrievals compared to the former standard workflow (45). In the authors' opinions, this efficient, practical approach can have an easy and widespread adoption.

A Computerized reminder system (MGH) can aid in sending notifications—messages, emails—to both the responsible physician for retrieval consideration and the patient for communication and preparation.

Since IVCFs result in a billing event (such as at Massachusetts General Hospital), the billing process can be used to trigger a dedicated system leading to patient follow-up (80).

Undoubtedly, the program is more efficient if based on a multicenter “Filter Registry”—which comprises patient electronic medical records. A Spanish multicenter real-life registry of retrievable vena cava filters (REFiVeC) (53) involved 15 major tertiary hospitals in facilitating close patient follow-up for prompt filter removal. They achieved a global retrieval rate of 76.9%, with an adjusted rate of 94.15% and no major complications. However, the total recovery rate in their registry might seem low compared to the CIRSE Registry (92% retrievals) (81), but presents similar or somewhat better results than the British Society of Interventional Radiology (BSIR) registry rate of about 65% (83% technical success out of 78% retrieval attempts) (40).

## Recommendations

An FDA study in 2013 evaluating the safety of implantation and removal of IVCFs in terms of risk vs. benefit profile advocated rIVCF retrieval between 29 and 54 days after placement, once the threat of PE tapers (82).

Directly binding the IVCF placement report with a timely scheduled visit is an easy and effective means of following the patient and boosting the quality control of the service provided. Informing the patient is a key part of the puzzle.

Incentive measures could also be kept in mind; IVCF retrieval rates could be part of the standardized metric in governmental surveys for the “best ranks.” In another approach, a much higher payment could be chosen for filter removal compared to filter placement only.

Advanced and more aggressive techniques would improve the retrieval rates, though with the expense of increased risk of complications (37, 51, 83). Therefore, all practitioners implanting IVCFs should acquire expertise in advanced retrieval techniques (84, 85). This can be accomplished through spotlighting teams, continuing medical education programs to approach the ideal rate of 100% successful retrievals in technically eligible patients. This verge should apply to all rIVCFs, regardless of dwell time; in fact, the concept that a filter of more than 6 months is “too risky to remove” is not a good reason for giving up (52). Setting up a national referral network of centers of excellence for IVCFs whose retrieval have failed at local institutions is another practical solution. Studies reported that different vendors' filter types or models do not have any significant inequality in retrievability (29, 35, 40, 54, 86–88).

## Conclusions

The current widespread use and “out of sight, out of mind” trend to IVCF placement and retrieval in practices demands to be revised and replaced with a more focused approach. More precise indications for insertion, more appropriate filter choice according to the indication, patient's clinical status, filter technical characteristics, and extra measures driving proper filter retrievalare strongly needed. The necessity of removing filters as soon as possible must be recognized in order to achieve the filter's maximum helping potential and avoid the possibility of long-term complications.

## Author Contributions

KR-K and DR contributed to writing the manuscript with input from all authors. SQ was involved in planning, revision, and supervising the project. All authors contributed to the article and approved the submitted version.

## Conflict of Interest

The authors declare that the research was conducted in the absence of any commercial or financial relationships that could be construed as a potential conflict of interest.

## Publisher's Note

All claims expressed in this article are solely those of the authors and do not necessarily represent those of their affiliated organizations, or those of the publisher, the editors and the reviewers. Any product that may be evaluated in this article, or claim that may be made by its manufacturer, is not guaranteed or endorsed by the publisher.
